# Blinatumomab in Ph+ B-ALL: present and perspectives

**DOI:** 10.18632/oncotarget.22071

**Published:** 2017-10-25

**Authors:** Cristina Papayannidis, Giovanni Martinelli

**Affiliations:** Cristina Papayannidis: Institute of Hematology and Medical Oncology “L. and A. Seràgnoli”, Department of Experimental, Diagnostic and Specialty Medicine, Bologna University School of Medicine, Bologna, Italy

**Keywords:** acute lymphoblastic leukemia, blinatumomab, minimal residual disease, philadelphia chromosome, tyrosine kinase inhibitors

In the last few decades, two innovative findings have revolutionized the clinical management of Acute Lymphoblastic Leukemia (ALL): the introduction of tyrosine kinase inhibitors (TKIs) in the treatment of Philadelphia positive (Ph+) patients, and the assessment of the crucial role of minimal residual disease (MRD) in the decision making of the most appropriate therapeutic path, both in Ph+ and Philadelphia negative (Ph-) populations. In Ph+ patients, TKIs led to a significant outcome improvement, mainly in terms of better tolerability, and reduction of death during induction rates, if compared with standard chemotherapy-based regimens [1]. Nevertheless, in this context, relapse still remains an urgent unmet clinical need for physicians, even after allogeneic hematopoietic stem cell transplantation (alloBMT), which still represents the gold standard procedure for young patients in first complete remission (CR). Emerging high throughput techniques have deeply clarified, from a biological point of view, the molecular mechanisms responsible for resistance or response loss after TKIs treatment, frequently represented by the occurrence of substitutions in the *ABL* kinase domain, particularly within the threonine 315 residue (T315I). However, relapse can also occur without detection of *BCR-ABL1* mutations, suggesting that there are kinase-independent pathways to leukemic cell survival and proliferation [2]. In such a dismal scenario, second or third generation TKIs as salvage therapy are able to offer only short remissions in the majority of the patients. Therefore, alternative approaches beyond kinase inhibition are strongly needed. In the field of immunotherapy, which currently represents the most promising area of development of innovative therapeutic strategies for tumours, Blinatumomab was the first compound that demonstrated both a good safety profile and a relevant antileukemic activity. Blinatumomab is a first-in-class bispecific T-cell engager (BiTE) antibody derived from a B-lineage specific antitumor mouse monoclonal antibody, that binds to both CD19 of B-cells and CD3 of T-cells. The tight binding and the close proximity to the CD19-positive B-cells and leukemic cells leads to non-major histocompatibility complex-restricted T-cell activation, polyclonal T-cell expansion and direct target cell killing. Administered by continuous infusion, Blinatumomab has proven to be a powerful therapeutic option in relapsed/refractory (R/R) ALL patients, both adult and paediatric, thanks to its strong efficacy and limited toxicity. In details, Blinatumomab is able to determine morphological CR rates ranging from 39% to 69% in Ph- R/R ALL adult patients (compared to 25% after second-line chemotherapy) with prolonged overall survival (OS) (Blinatumomab median OS, 7.7 months vs chemotherapy, 4.0 months) [3]. Furthermore, in comparison to conventional cytotoxic second-line approaches, Blinatumomab has a more favourable safety profile. The main adverse event is represented by cytokine-release syndrome (CRS) that can be managed by interruption and/or the application of steroids or Tocilizumab. Another typical complication is the occurrence of neurological side effects, such as seizures and encephalopathy, usually reversible after application of steroids and/or withdrawal of drug infusion. In Ph^+^ ALL patients, with R/R disease, who progressed after or were intolerant to a second-generation or later TKI, the Alcantara phase II, single-arm, multicenter clinical trial [4] investigated the role of Blinatumomab, administered in 28-day cycles by continuous intravenous infusion. The protocol aimed at assessing CR or CR with partial hematologic recovery (CRh) rates, during the first two cycles of treatment. Secondary endpoints were represented by MRD response, rate of alloBMT, relapse free survival (RFS), OS and toxicity assessment, in terms of adverse events (AEs). Of 45 patients, 16 (36%; 95% CI, 22% to 51%) achieved CR/CRh during the first two cycles, including four of 10 patients with the T315I mutation; 88% of CR/CRh responders achieved a complete MRD negativity. Seven responders (44%) proceeded to alloBMT, including 55% (6 of 11) of transplantation-naïve responders. Median RFS and OS were 6.7 and 7.1 months, respectively. The most frequent AEs were pyrexia (58%), febrile neutropenia (40%), and headache (31%). Three patients developed CRS (all grade 1 or 2), and three patients had grade 3 neurologic events, one of which (aphasia) required temporary treatment interruption. No grade 4 or 5 neurologic events occurred. Taken together, these data showed a relevant antileukemic activity of Blinatumomab as single-agent in a vey poor risk ALL population, confirming the promising results emerged from the experience in Ph negative setting, in terms of safety and efficacy. Nevertheless, many questions on this topic are still open. First, at least four mechanisms of drug resistance have been hypothesised, and others are under investigation. Reduction of CD19 expression [5], lymphoid to myeloid lineage switch [6], induction of checkpoint molecules / T-cell exhaustion [7] and impaired T reg/T eff ratio [8] may play a role. Secondly, the identification of biomarkers for response prediction and toxicity may represent a useful tool for physicians, in order to identify the patients who may potentially benefit from this approach. Moreover, in terms of optimization of the therapeutic strategy of Ph+ ALL patients, the most appropriate combination approach with Blinatumomab needs to be explored. In conclusion, data published by Martinelli et al confirmed the efficacy of a single agent approach in relapsed/refractory Ph+ ALL patient, highlighting the safety and feasibility of the infusion of a compound with an innovative mechanism of action and a new way of administration. Nevertheless, long term cure of such an aggressive disease is unfortunately still far away, due to a high relapse rate, even after alloBMT. In this scenario, we strongly believe that a deeper understanding of the above mentioned biological issues would open the way for a potential “chemo-free” approach, at least in the elderly population, based on the combination of Blinatumomab and second/third generation TKIs.
